# P109 Not all weakness is neurological: Juvenile dermatomyositis presenting as motor regression with normal CK in a 3-year-old child

**DOI:** 10.1093/rap/rkag113.139

**Published:** 2026-11-03

**Authors:** Suchika Garg, Georgios Eleftheriou, Laura Hyrapetian

**Affiliations:** Royal London Hospital, London, United Kingdom; Royal London Hospital, London, United Kingdom; Royal London Hospital, London, United Kingdom

## Abstract

**Introduction:**

Juvenile dermatomyositis (JDM) is the most common inflammatory myopathy of childhood, characterized by proximal muscle weakness and characteristic cutaneous manifestations. Diagnosis can be challenging when classical laboratory features such as markedly elevated creatine kinase (CK) are absent. We report a young child presenting initially to neurology services with progressive motor regression following a viral illness, in whom subtle dermatological findings ultimately led to the diagnosis of JDM.

**Case description:**

A previously healthy 3-year-10-month-old British Bangladeshi girl developed progressive weakness following a febrile viral illness at age 3 years 2 months. Over eight months she experienced increasing fatigue, frequent falls, loss of previously acquired motor skills, inability to climb stairs, difficulty rising from the floor, impaired sitting balance, and progressive upper limb weakness affecting dressing and use of cutlery.

Extensive neurological and metabolic investigations were undertaken. MRI brain and spine were normal. Spinal muscular atrophy testing, metabolic screening, plasma amino acids, urine organic acids, and acetylcholine receptor and MuSK antibodies were negative. Whole genome sequencing was sent which showed no clinically relevant variants found. CK remained within the normal range (118–170 IU/L). Neurophysiological studies demonstrated normal sensory nerve conduction studies, with symmetrically reduced compound motor action potential (CMAP) amplitudes and no evidence of conduction slowing. Electromyography showed spontaneous activity and myopathic features, supporting an inflammatory myopathic process

On detailed questioning, her mother reported a fluctuating facial rash present for approximately two years which was previously managed as eczema with emollients. Examination demonstrated marked proximal muscle weakness with a positive Gower’s sign, myopathic gait, inability to lift her head from supine, mild knee synovitis, malar erythema with perioral and perinasal sparing, hyperpigmentation over the eyelids, metacarpophalangeal joints and knees suggestive of heliotrope rash and Gottron-like changes.

Laboratory investigations showed elevated ANA (1:2560, speckled, homogenous), ESR (35 mm/hr), AST (64 U/L) and LDH, with normal CK. Electromyography demonstrated spontaneous activity and myopathic features consistent with inflammatory myopathy. Myositis-specific antibody (Anti-Tif1y) was positive.

She is currently under physiotherapy rehabilitation program and on prednisolone and methotrexate for management of the disease symptoms.

**Discussion:**

This case highlights an atypical presentation of juvenile dermatomyositis in a very young child, where progressive motor regression and normal CK initially directed investigations towards neurological, genetic and metabolic causes. The patient followed a diagnostic pathway from general paediatrics to neurology and finally rheumatology, illustrating the challenges of recognising inflammatory myopathy when classical laboratory features are absent.

Several important clinical clues emerged during repeated assessment. Profound neck flexor weakness, characteristic skin changes, elevated AST and LDH, and abnormal nailfold capillaries were highly suggestive of juvenile dermatomyositis despite a normal CK. Neurophysiology was pivotal in redirecting the diagnostic pathway towards an inflammatory myopathy, demonstrating myopathic features without evidence of neuropathy.

In retrospect, recognition of the combination of rash and proximal muscle weakness could have prompted earlier consideration of inflammatory myopathy. As MRI brain and spine were being performed, MRI of the thighs with myositis-sensitive sequences may have provided earlier evidence of muscle inflammation. The case also highlights the importance of close collaboration between paediatrics, neurology and rheumatology when evaluating progressive weakness in childhood.

**Key learning points:**

• Juvenile dermatomyositis should be considered in children presenting with progressive proximal muscle weakness and motor regression, even when CK is normal.

• Juvenile dermatomyositis should be considered in any child presenting with progressive proximal weakness, falls and motor regression, even when initial investigations are non-diagnostic.

• A normal CK does not exclude active inflammatory myopathy; AST, ALT,LDHand other muscle enzymes may provide important evidence of muscle inflammation.

• The combination of rash and proximal muscle weakness shouldimmediatelyprompt consideration of inflammatory myopathy.

• Profound neck flexor weakness is an important clinical sign and may strongly suggest juvenile dermatomyositis.

• Progressive motor regression in childhood is not always neurological,geneticor metabolic; rheumatological diseasesremainimportant differentials.

• Neurophysiology can be invaluable in distinguishing inflammatory myopathy from neuropathic and neurodegenerative disorders when CK is normal.

• MRI brain and spine may be normal in juvenile dermatomyositis; muscle MRI with dedicated myositis sequences should be considered when inflammatory myopathy is suspected.

• Nailfold capillaroscopy is a simple bedside investigation that can provide a crucial clue to an underlying connective tissue disease and should be routinely considered in suspected JDM.

• Careful and repeated skin examination may reveal subtle heliotrope, Gottron-like or malar changes that are initially overlooked.

• Multidisciplinary collaboration between general paediatrics, neurology and rheumatology is essential to minimise diagnostic delay in children with progressive weakness.

